# Preoperative incidence and locations of deep venous thrombosis (DVT) of lower extremity following ankle fractures

**DOI:** 10.1038/s41598-020-67365-z

**Published:** 2020-06-24

**Authors:** Zixuan Luo, Wei Chen, Yansen Li, Xiaomeng Wang, Weili Zhang, Yanbin Zhu, Fengqi Zhang

**Affiliations:** 1 Department of Foot and Ankle Surgery, The 3rd Hospital of Hebei Medical University, Shijiazhuang, 050051 Hebei People’s Republic of China; 2 Department of Orthopaedic Surgery, The 3rd Hospital of Hebei Medical University, Shijiazhuang, 050051 Hebei People’s Republic of China; 3 Key Laboratory of Biomechanics of Hebei Province, Shijiazhuang, 050051 Hebei People’s Republic of China

**Keywords:** Risk factors, Trauma

## Abstract

This retrospective study aimed to investigate the preoperative incidence and locations of deep venous thrombosis (DVT) in patients undergoing surgeries for ankle fractures and identify the associated risk factors. From January 2016 to June 2019, 1,532 patients undergoing surgery of ankle fractures were included. Their inpatient medical records were inquired for data collection, including demographics, comorbidities, injury-related data and preoperative laboratory biomarkers. DVT of bilateral lower extremities was diagnosed by routine preoperative Doppler examination. Univariate analyses and multivariate logistic regression analyses were used to identify the independent risk factors. Totally, 98 patients had a preoperative DVT, indicating an incidence rate of 6.4%. A total of 164 clots for 6 veins were found, representing an average of 1.7 for each patient. The detailed DVTs involving veins were as follows: 2 in femoral common vein, 7 in superficial femoral vein, 2 in deep femoral vein, 16 in popliteal vein, 49 in posterior tibial vein, and 88 in peroneal vein. In the multivariate model, 5 risk factors were identified to be associated with DVT, including age (10-year increase), gender, lower ALB level, reduced LYM count and elevated D-dimer level. There was a tendency for diabetes mellitus to increase the risk of DVT, although there was no statistical significance (p = 0.063). These epidemiologic data on DVT may help counsel patients about the risk of DVT, individualized risk assessment and accordingly the risk stratification.

## Introduction

Ankle fracture is a common fracture type, representing 7.6% of all adult fractures^1^. Because of the special anatomical features, most of the ankle fractures involve the articular surface to varying degrees. The relatively thin skin and less soft tissue coverage more likely result in localized swelling and blood hypercoagulability. Surgical interventions are commonly delayed until the soft tissue envelop is deemed safe, which, therefore, leads to a prolonged duration of extremity elevation and limited mobility. These constitute the pathophysiological basis for preoperative thrombosis genesis.

Deep venous thrombosis (DVT) is a significant cause of morbidity and is associated with increased risk of pulmonary emboli and mortality^2,3^. By far, orthopaedics studies on DVT mainly focus on hip or knee joint arthroplasties^4,5^, the major trauma (hip fracture, spinal injury and others)^6–8^ or a specific population (e.g., elderly, diabetes)^7^. Specified at ankle trauma, however, there is still lack of epidemiologic data. In most cases, it is difficult to conduct multivariate analyses due to low incidence of DVT and the small sample size, especially after routine prophylactic use of thromboprophylaxis agents. On the other hand, some studies used national database to address this issue^9–11^, but the relatively few variables included and heterogeneity among participants, variation in DVT diagnosis and data collection might be the new concerns. Moreover, in some studies, authors did not distinguish between preoperative and postoperative thrombosis^9^, which, therefore, provided less targeted reference value in prevention of DVT.

Applicability of these results from studies which were specified at other fractures or from studies with small samples or fewer variables included might be poor; also, use of these results might result in a misdirected guidance in constructing a risk prediction model for DVT. Given that, we conducted this study, using data from inpatient medical records and laboratory tests, to evaluate the epidemiologic characteristics of preoperative DVT.

## Methods

This retrospective study was conducted in accordance with Strengthening the Reporting of Cohort Studies in Surgery (STROCSS) guideline. The ethics committee of the 3rd Hospital of Hebei Medical University approved this study, and waived the requirement for informed consent due to the anonymous nature of the data.

### Inclusion and exclusion criteria

Patients who were admitted between January 2016 to June 2019 due to acute ankle fractures were included in this study. Inclusion criteria were patients aged 18 years or older, fresh ankle fracture (≤ 3 weeks after injury), and complete medical records. Patients with pathological (metastatic), old fracture (> 3 weeks after injury), open fracture, multiple fractures, or incomplete medical records, use of anticoagulant or antithromboembolic agents due to chronic comorbidities, were excluded.

### Diagnosis of DVT

Guideline for the Diagnosis and Treatment of Deep Vein Thrombosis (3rd edition) was used to diagnose DVTs^12^. Before the operation, routine Doppler ultrasound examination of vein of bilateral lower extremities was performed to detect DVT based on the signs of deep venous lumen obstruction or filling defect. The involving veins included femoral common vein, superficial femoral vein, deep femoral vein, popliteal vein, posterior tibial vein, anterior tibial vein and peroneal vein. Superficial or intermuscular vein thrombosis (soleal or gastrocnemius vein thrombosis) was excluded, due to its less clinical significance^13,14^.

### Data collection

Inpatient electronic medical record system, radiographic image and operation report were retrieved for data collection. The data included demographics (age, gender, residence place), lifestyles (smoking, alcohol consumption), comorbidities (body mass index (BMI), hypertension, diabetes, chronic heart disease, any surgery history and allergies to any medications self-reported by patients) and injury-related data (fracture location, injury mechanism), presence or absence of dislocation and American Society of Anesthesiologists (ASA) grade. The BMI (kg/m^2^) was divided into normal (18.5–23.9), underweight (< 18.5), overweight (24.0–27.9) and obesity (≥ 28.0), based on the criteria for the Chinese population^15^. An injury caused by a fall from standing height was defined to be low-energy injury, and others including fall from a height, motor accidents et al. were defined as high-energy injury.

Preoperative laboratory tests included measurements of total protein (TP) level, albumin (ALB) level, fasting blood glucose (FBG) level, red blood cell (RBC) count, white blood cell (WBC) count, neutrophile (NEUT) count, lymphocyte (LYM) count, neutrophile/lymphocyte rate (NLR), hemoglobin (HGB) level, haematocrit (HCT), platelet (PLT), red blood cell distribution width (RDW), serum total cholesterol (TC) level, triglyceride (TG) level, low density lipoprotein (LDL-C) level, high density lipoprotein (HDL-C) level, very low-density lipoprotein (VLDL) level and D-dimer level. For patients who had multiple laboratory tests before when DVT was detected, the closest laboratory test was chosen for data analysis.

### Statistical analysis

Student-*t* test or Mann Whitney-*U* test was used to evaluate the difference of continuous variables, based on the normality of data distribution. Chi-square or Fisher's exact test was used to evaluate the difference of categorical variables. Multivariate logistics regression analysis was performed to identify the risk factors independently associated with DVT, using the stepwise backward elimination method. Variables with p < 0.10 were retained in the final model, and odd ratio (OR) with its 95% confidence interval (95% CI) was to indicate the correlation strength. Two-tailed p < 0.05 was set as the statistical significance level. The goodness of fit of the final multivariate model was evaluated by Hosmer–Lemeshow (H–L) test, with p > 0.05 representing the acceptable result. All the analyses were conducted by SPSS23.0 software (IBM, Armonk, New York, USA).

## Results

During the study window, 1532 patients (1555 ankle fracture) were included, comprising 888 males and 644 females, with 43.0 years in average (Standard deviation, SD, 15.1; range, 18–95; median, 43.0). There were 712 (45.8%) unimalleolar fracture (712/1,555), 347 (22.3%) cases of bimalleolar fractures and 496 (31.9%) cases of trimalleolar fractures. Among all fractures, 284 (18.3%) were accompanied by dislocation or subluxation. The total hospitalization stay was 16.6 days (Sd, 14.0; median, 14; range, 2 to 341).

Totally, 98 were diagnosed to have preoperative DVT, indicating an incidence rate of 6.4%. Except for anterior tibial vein, 164 thrombi were found in 6 veins, representing an average of 1.7 (range, 1 to 6) for each patient (164/98). In 13 (13.3%) patients, DVT involved both the injured and non-injured limbs. In 18 (18.4%) patients DVT involved only the non-injured limb. In detail, DVTs involved femoral common vein in 2 patients, superficial femoral vein in 7, deep femoral vein in 2, popliteal vein in 16, posterior tibial vein in 49, and peroneal vein in 88 patients. The median time of diagnosis of DVT was 2 days [interquartile range (IQR), 1 to 2] after injury, ranging from 0 to 14 days; 77.6% (76/98) were diagnosed within 2 days and 94.9% (93/98) were diagnosed within 7 days after the injury.

There was significant difference between DVT and non-DVT patients regarding gender, age (both in continuous and categorical variable), past diabetes mellitus, past any surgery, injury mechanism, ASA grade, TP, ALB, FBG, LYM, RBC count, HGB level, HCT, and D-dimer level (Table 1). Patients with DVT had to stay for a significantly prolonged 6.4 days than those with DVT (22.6 vs. 16.2 days) (Table 1).

Initially, 18 variables were entered into multivariate model, including 15 variables tested significant in the univariate analyses together with cigarette smoking, hypertension and elevated LDL-C level (all p < 0.10). In the final model, age (in 10-year increase), male gender, ALB < 35 g/L, LYM count < 1.8 × 10^9^/L and D-dimer > 0.3 mg/L (Table 2). Diabetes mellitus was found to have a tendency towards to increase the risk of DVT, but that was not statistically significant (p = 0.063). The goodness of fit of the final multivariate model was adequate (X^2^ = 2.256, p = 0.972; Nagelkerke R^2^ = 0.104).

**Table 1:** Univariate analyses of risk factors associated with DVT following ankle fracture surgeries.

Variables	Number (%) of DVT (n = 98)	Number (%) of non-DVT (n = 1,434)	*p*
**Gender (male)**	70 (71.4)	818 (57.0)	0.005
**Age (years)**	48.4 ± 14.4	43.6 ± 15.1	0.003
18–44	36 (36.7)	769 (54.1)	0.004
45–64	47 (48.0)	526 (36.4)	
65 or older	15 (15.3)	139 (9.7)	
**Living place**			0.824
Rural	57 (58.2)	791 (55.2)	
Urban	41 (41.8)	641 (44.8)	
**BMI (kg/m**^**2**^)	26.0 ± 3.0	25.9 ± 4.1	0.872
18.5–23.9	23 (28.9)	419 (29.2)	
< 18.5	5 (5.3)	25 (1.7)	
24.0–27.9	47 (47.4)	623 (43.4)	
≥ 28.0	23 (18.4)	367 (25.6)	
**Cigarette smoking**	22 (22.4)	217 (15.1)	0.053
**Alcohol consumption**	27 (27.6)	395 (27.5)	0.999
**Diabetes mellitus**	20 (20.4)	185 (12.9)	0.035
**Hypertension**	19 (19.4)	189 (13.2)	0.083
**Chronic heart disease**	4 (2.7)	66 (4.8)	0.259
**History of allergies to any medications**	15 (15.3)	207 (14.4)	0.813
**History of any surgery**	21 (21.4)	113 (7.9)	< 0.001
**Injury mechanism (high-energy)**	38 (38.8)	352 (24.5)	0.002
**Fracture location**			0.893
Unimalleolar	46 (46.9)	667 (45.8)	
Bimalleolar	23 (23.5)	326 (22.3)	
Trimalleolar	29 (29.6)	464 (31.8)	
**Accompanied dislocation or subluxation**	24 (24.5)	262 (18.4)	0.108
**Total hospital stay**	22.6 ± 15.5	16.2 ± 14.6	< 0.001
**ASA class**			0.030
I	13 (13.3)	250 (17.4)	
II	66 (67.3)	1,028 (71.7)	
III or above	19 (19.4)	156 (10.9)	
**TP (< 60 g/L)**	34 (34.7)	253 (17.6)	< 0.001
**ALB (< 35 g/L)**	27 (27.6)	178 (12.4)	< 0.001
**FBG (> 6.1 mmol/L)**	42 (42.9)	405 (28.2)	0.002
**TC (> 5.2 mmol/L)**	41 (41.8)	518 (36.1)	0.256
**TG (> 1.7 mmol/L)**	14 (14.3)	274 (19.1)	0.237
**LDL-C (> 3.37 mmol/L)**	12 (12.2)	256 (17.9)	0.157
**HDL-C (< 1.1 mmol/L)**	43 (43.9)	547 (38.1)	0.259
**VLDL (> 0.78 mmol/L)**	14 (14.3)	266 (18.5)	0.291
**WBC (> 10 × 10**^**9**^**/L)**	24 (24.5)	376 (26.2)	0.706
**NEUT (> 6.3 × 10**^**9**^**/L)**	49 (50.0)	607 (42.3)	0.138
**LYM (< 1.1 × 10**^**9**^**/L)**	38 (38.8)	285 (19.9)	< 0.001
**RBC (< lower limit)**	38 (38.8)	281 (19.6)	< 0.001
**HGB (< lower limit)**	34 (34.4)	279 (19.5)	< 0.001
**HCT (< lower limit)**	58 (59.2)	576 (40.2)	< 0.001
**PLT (> 300 × 10**^**9**^**/L)**	24 (24.5)	258 (18.0)	0.108
**PDW (> 18.1%)**	14 (14.3)	133 (9.3)	0.103
**RDW (> 16.5%)**	5 (5.1)	54 (3.8)	0.506
**D-dimer (> 0.3 mg/L)**	72 (73.5)	851 (59.3)	0.006

BMI, body mass index; ASA, American Society of Anesthesiologists; RBC, red blood cell, reference range: Female, 3.5–5.0 × 10^12^/L; males, 4.0–5.5 × 10^12^/L. HGB, hemoglobin, reference range: Females, 110–150 g/L; males, 120–160 g/L; FBG, fasting blood glucose; HCT, haematocrit, 40–50%; WBC, white blood cell; NEUT, neutrophile; LYM, lymphocyte; PLT, platelet, 100–300 × 10^9^/L; TP, total protein; ALB, albumin; RDW, red cell distribution width; PDW, platelet distribution width; TC, total cholesterol; TG, triglyceride, LDL-C, low density lipoprotein; HDL-C, high density lipoprotein; VLDL, very low-density lipoprotein.

**Table 2:** Multivariate analyses of risk factors associated with preoperative DVT in ankle fracture surgeries.

Variable	OR and 95% CI	*p* value
Age (increase of every 10 year)	1.16 (1.00 to 1.34)	0.047
Diabetes mellitus	1.68 (0.97 to 2.92)	0.063
Gender (male vs. female)	2.35 (1.46 to 3.77)	< 0.001
ALB < 35 g/L	2.06 (1.25 to 3.39)	0.005
LYM count (< 1.8 × 10^9^/L)	1.92 (1.22 to 3.00)	0.005
D-dimer > 0.3 mg/L	1.65 (1.02 to 2.68)	0.042

DVT, deep venous thrombosis; OR, odd ratio; CI, confidence interval; LYM, lymphocyte.

## Discussion

It is of utmost important to understand the epidemiologic characteristics of DVTs and use the risk factors for individualized risk assessment and risk stratification. Also, it is important to distinguish between preoperative and postoperative DVT and know well about where and when DVT is more likely to develop. In this study, we found that the incidence rate of DVT in ankles before operation was 6.4%. We also determined the locations and timing of DVTs, and identified several independent risk factors, including age, gender, ALB level, LYM count and D-dimer.

There is a great variation in the reported incidence of DVT associated with ankle fractures^9,10,16^. This is due to study design, variation in diagnostic criteria and heterogeneity of included patients. Duan et al.^16^ retrospectively evaluated 344 cases of foot and ankle fracture and found the preoperative incidence rate of 13.7%. However, in their study, authors did not exclude plexus venosus leg muscle thrombosis which had less clinical significance, and these accounted for 51% of all DVTS. In two large-sample studies with data available from national databank, authors reported the incidence of DVT of 0.05% to 0.28% in foot and ankle trauma, but authors did not distinguish between preoperative and postoperative DVT, not isolate the ankle fractures from the overall fractures^10^ or only specified DVT or pulmonary embolism within 90 days of surgery^9^. These results might be unreasonable to guide clinical practices and less generalizable to others. In a single-center study, authors reported the comparable rate as ours (6.4%), that is 5.1% for preoperative DVTs following isolated ankle fractures^17^. In other studies, authors focused on the symptomatic venous thromboembolism following surgery of ankle fractures or other fractures^18,19^, but we were unable to obtain the adequate data to perform the statistical analyses due to the retrospective design and the very limited number of symptomatic cases.

In this study, over 80% of the DVTs involved the injured extremity or the both extremities, and distal (tibial and peroneal veins) DVTs took a predominant proportion (83.5%), which was in range of the figures reported in literature^13,16,20^. We also observed the trend towards a relatively higher risk of DVT with the prolonged duration from fracture that most DVTs were diagnosed within 2 days after injury (94.9%). This may be explained by the previous conclusion that the blood coagulation was highest in 1–4 days after trauma^17^. From our experience and findings from literature, it is most possible that patients who experience the delay time from fracture to surgery are right in dynamic peak of blood hypercoagulability.

In literature, age as a well-known associated risk factor for DVT was extensively discussed. In this study, patients with DVT were 4.8 years older than those without DVT, and each increment of 10-years was associated with increased 16% risk of DVT. Consistent with ours, Shibuya et al.^10^ found a converted 20% increased risk of acute DVT with increment of every 10-year in their study of 75,654 patients with foot and ankle trauma. However, in our multivariate model, age as a categorical variable was not tested to be significant when it was divided into 3 groups based on criteria proposed by WHO. This was inconsistent with the previous findings of Chinese and US researches that 40 years^16^, and 50 and 75 years^9^ used as cut-off values were significantly associated with increased risk of DVT following ankle trauma. This might indicate that racial, ethnical or individual variation in vascular aging and sensitivity to thrombosis formation should be also the deserving contributing factors, which, however, required future cohort study to investigate.

The relationship between gender and DVT occurrence remains in controversy. In a study of 159 cases of calcaneal fractures, Williams et al.^21^ observed a significantly increased risk of preoperative DVT in males (14.0%) than that of in females (6.5%). Similarly, Duan et al.^22^ found the male patients had a significantly higher preoperative incidence of DVT (including the intermuscular DVT) following surgeries of elderly hip fractures than that in females (42.7% vs. 28.9%). In most other studies of trauma, no significant relationship between gender and DVT was found; and specified at ankle trauma, our statistically significant finding was firstly reported. The further studies should focus on exploring the underlying mechanism or elimination of confounding effects that more likely related to gender (estrogen level, lifestyles, or fracture severity).

In elective major orthopaedic surgeries such as arthroplasties or orthopaedic trauma (hip fracture), diabetes was a well-known risk factor for DVT^23–25^, which was related to the diabetic vascular endothelial injury. Specified at ankle fracture, Yi et al.^17^ found preoperative incidence of DVT of 5.1% in 2,347 patients with an isolated ankle fracture and identified diabetes as independent risk for DVT after adjustment for BMI, smoking history and activity level. Consistent with that, we found diagnosis of diabetes was associated with 1.68-fold increased risk of DVT, although it was not significant statistically (p = 0.063). Similarly, the measurement of FBG level was not tested to be significant in the multivariate model, although significant in univariate analysis (p = 0.002). The broader definition of elevated FBG as 6.1 mmol/L might be an explanation, and the post-traumatic stress response leading to FBG level elevation should be also an important reason. Despite, perioperative strict control of glycemia and optimized diabetes conditions are still critical, because they are related to many comorbidities or complications^26^.

In this study we also identified 3 biomarkers as independent risk factors for DVT, including ALB level, LYM count and D-dimer level, and they had been extensively discussed in the studies of orthopaedics or other subspecialties^27–29^, or specified at ankle trauma^16^. Duan et al.^16^ found the risk of DVT was increased by 3.3-fold in patients with D-dimer level > 0.3 mg/L than that < 0.3 mg/L, a stronger association than ours (OR, 1.65). The declining ALB level and reduced LYM count are more likely indication of a low immune and systematic inflammation status, but the stress effects from trauma cannot be ruled out^30^. The elevation of D-dimer level in plasma reflects the secondary increased fibrinolytic activity, indicating the hypercoagulability and fibrinolytic hyperactivity in vivo, which is a marker of thrombosis or solution and is of great value in diagnosis of thrombotic events. These biomarkers are a part of blood routine examination, readily obtained and inexpensively, and could be used as auxiliary predictive or diagnostic factors to improve the DVT detection rate.

The advantage of this study was inclusion of a large sample and multiple potential variables involving demographics, injury and preoperative laboratory tests. Despite, several limitations should be noted. First, retrospective design was the internet limitation, which might affect the accuracy in data collection, especially the selt-reported comorbidities. Second, as with every other multivariate analysis, this study could not include all the potential influential factors, such as duration of immolisation of the injured limb^13^. Third, for some uncommon medical conditions or comorbidities such as chronic liver disease or nephrosis which have significant effects on metabolism of inflammatory factors or immune cells, we are unable to investigate their association with DVT due to the limited number of sample. Fourth, this study was a single-center teaching study with patients having a lower BMI and most patients were operated after 24 h after admission, therefore, the results might be less generalisable to European patients and others.

In conclusion, the incidence rate of preoperative DVT was 6.4% in ankle fractures. About 80% of DVTs were detected within 2 days after injury and 95% within one week. The distal DVT took a predominant proportion (83.5%). Age (10-year increase), gender, lower ALB level, reduced LYM count and elevated D-dimer level were identified to be associated with DVT. These epidemiologic data may help counsel patients about the risk of DVT, individualized risk assessment and the accordingly risk stratification.

## Data Availability

All the data will be available upon motivated request to the corresponding author of the present paper.
